# Outcomes of Isolated Severe Blunt Splenic Injury

**DOI:** 10.1001/jamanetworkopen.2025.33266

**Published:** 2025-09-23

**Authors:** Wei Huang, Caitlyn Braschi, Feifei Jin, Meghan Lewis, Demetrios Demetriades

**Affiliations:** 1 Division of Trauma and Acute Care Surgery, Department of Surgery, Los Angeles General Medical Center, Los Angeles, California; 2 Peking University People’s Hospital, Trauma Center, Beijing, China

## Abstract

**Importance:**

Management of blunt splenic injury is evolving toward wider use of nonoperative approaches for splenic salvage, and splenic angioembolization (SAE) is being considered even in patients with hypotension on admission. Research is needed to understand the outcomes of these evolving management strategies.

**Objective:**

To compare outcomes of the 3 major treatments approaches for splenic injury.

**Design, Setting, and Participants:**

A retrospective cohort study was performed using the American College of Surgeons Trauma Quality Improvement Program (ACS-TQIP) database from January 1, 2017, to December 31, 2022. The database collects injury data from more than 815 trauma centers in the US. Adults with isolated, severe (Abbreviated Injury Scale score ≥3) blunt splenic injury were identified. Isolated splenic injury was defined by the absence of other intra-abdominal injury and any other major associated injuries with an Abbreviated Injury Scale score of 3 or higher. Data analysis was performed from September to December 2024.

**Exposure:**

Open splenectomy (OS) vs SAE vs observation.

**Main Outcomes and Measures:**

The primary outcomes were mortality and any complication. Outcomes were compared using multivariable Cox proportional hazards regression analyses.

**Results:**

A total of 7567 patients (median [IQR] age, 36 [25-55] years; 4901 men [64.8%]) were studied, including 1499 (19.8%) in the OS group, 1547 (20.4%) in the SAE group, and 4521 (59.7%) in the observation group. In multivariable analysis, there was no difference in mortality in the overall cohort or in subgroups. Morbidity was significantly lower in the SAE (odds ratio [OR], 0.61; 95% CI, 0.45-0.81; *P* < .001) and observation (OR, 0.71; 95% CI, 0.55-0.92; *P* = .01) groups compared with the OS group. Among patients with hypotension, there was no mortality difference, but shorter hospital length of stay was found in the SAE (β = −1.44; 95% CI, −1.79 to −1.09; *P* < .001) and observation (β = −1.41; 95% CI, −1.73 to −1.09; *P* < .001) groups. Compared with initial OS, morbidity was higher for patients in whom SAE (OR, 5.39; 95% CI, 3.39-8.57; *P* < .001) and observation (OR, 1.95; 95% CI, 1.44-2.64; *P* < .001) failed, and hospital length of stay was longer for these groups as well (β = 2.50; 95% CI, 1.27-3.73; *P* < .001 and β = 0.71; 95% CI, 0.07-1.35; *P* = .03, respectively).

**Conclusions and Relevance:**

In this retrospective cohort study, nonoperative management (SAE or observation) was associated with favorable outcomes when compared with OS in isolated severe blunt splenic injury, even in patients with hypotension on admission. Failure of nonoperative management, however, risked higher morbidity without associated increase in mortality. With careful patient selection, splenic salvage may be possible and preferred even in severely injured patients.

## Key Points

**Question:**

What is the optimal approach to managing severe splenic injuries?

**Findings:**

In this cohort study of 7567 patients, nonoperative management (angioembolization or observation) was associated with favorable outcomes when compared with surgery in isolated severe blunt splenic injury, even in patients with hypotension on admission.

**Meaning:**

This study’s results suggest that with careful patient selection, nonoperative management and splenic salvage may be possible and preferred even in high-grade splenic injuries.

## Teaser

This cohort study compares outcomes of splenic angioembolization, open splenectomy, and observation in patients with severe isolated blunt splenic injury.

## Introduction

The management of blunt splenic injury has evolved in recent decades, with a trend toward splenic salvage.^1^ The spleen has important immunologic functions, particularly in guarding against encapsulated bacteria. Patients undergoing splenectomy are at risk of infectious and noninfectious complications.^2,3,4,5^

Splenic angioembolization (SAE) has become an important adjunct for nonoperative management in blunt splenic trauma.^6^ SAE increases rates of splenic salvage.^7,8,9^ SAE carries associated risks, such as venous thromboembolism, infarction, and failure of nonoperative management.^10,11,12^ Furthermore, the long-term immune function of the spleen after SAE is not yet fully understood.^13^

Many retrospective studies^5,25,26^ have been published concerning blunt splenic injury, with most comparing operative management with observation in hemodynamically stable patients or comparing SAE with observation in hemodynamically unstable patients. A recent systematic review found that there have been no published studies comparing the outcomes of all 3 treatment strategies across both hemodynamically stable and unstable patients.^5^ The aim of this study was to perform a comprehensive comparison of outcomes of the 3 therapeutic modalities for severe isolated blunt splenic injury. To eliminate other variables that could complicate the interpretation of the outcomes, the study included only isolated severe splenic injuries.

## Methods

### Data Source

A retrospective cohort study was performed using the American College of Surgeons Trauma Quality Improvement Program (ACS-TQIP) database from January 1, 2017, to December 31, 2022. The ACS-TQIP database collects injury data from more than 815 trauma centers in the US. The study was approved by the institutional review board of the University of Southern California. The Strengthening the Reporting of Observational Studies in Epidemiology (STROBE) guidelines were used for design and reporting of this study.

### Patient Selection and Data Collection

Adult patients (aged ≥16 years) with isolated severe blunt splenic injury were included. In the ACS-TQIP database, race is self-reported by patients or their family members; these data are included because race may act as a potential confounder for the outcomes. Severe splenic injury was defined as an Abbreviated Injury Scale (AIS) score equal to 3 or more. Isolated splenic injury was defined by the absence of other intra-abdominal injuries and any other major associated injuries. Patients were excluded if they met any of the following criteria: AIS score of 3 or greater of any extra-abdominal region, severe nonsplenic intra-abdominal solid organ injury (AIS score ≥3), abdominal hollow viscus injury, named abdominal vascular injury, transferred from another facility, died in the emergency department, had a hospital length of stay (HLOS) of 24 hours or less, left against medical advice, underwent laparoscopic surgery or splenic repair (*International Statistical Classification of Diseases and Related Health Problems, Tenth Revision *[*ICD-10*] codes 07BP3ZZ, 07BP4ZZ, 07QP0ZZ, 07QP3ZZ, 07QP4ZZ, and 07TP4ZZ). Patients with missing data were excluded. Primary management was classified as operative splenectomy (OS) (*ICD-10* codes 07BP0ZZ and 07TP0ZZ), splenic angioembolization (SAE) (*ICD-10* codes 04L43DZ, 04L43ZZ, 04V43DZ, and 04V43ZZ), or observation. Observation was defined as no OS or SAE within 12 hours of hospital admission. Failure was defined as the need for OS or SAE after primary management.

Primary outcomes were mortality and any complication. Secondary outcomes included specific complications (acute kidney injury, acute respiratory distress syndrome, cardiac arrest, unplanned intubation, unplanned visit to operating room, ventilator associated pneumonia [VAP], venous thromboembolism, and surgical site infection), failure of primary management, HLOS, intensive care unit length of stay (ICULOS), and ventilator days.

### Statistical Analysis

Continuous variables with normal distributions were presented as means (SDs) and analyzed using a 2-tailed, unpaired *t* test. Nonnormally distributed continuous variables were reported as medians (IQRs) and compared using the Mann-Whitney *U* test. Categorical variables were expressed as numbers (percentages), with their statistical significance determined by the χ^2^ test or Fisher exact test. For multivariable analysis, all variables with *P* < .20 in univariable analysis were included. Data analysis was performed from September to December 2024.

#### Main Analysis

Both univariable and multivariable Cox proportional hazards regression analyses were conducted to assess the risk associated with the SAE and observation groups compared with the OS group; hazard ratios (HRs) and 95% CIs were calculated. For other binary outcomes, univariable and multivariable logistic regression analyses were used to evaluate risks, with odds ratios (ORs) and 95% CIs calculated. For continuous outcome variables, univariable and multivariable linear regression analyses were used to explore differences among groups, yielding regression coefficients (β) and 95% CIs. Model 1 was adjusted for age; model 2 was adjusted for age, race, body mass index, payment, hospital bed size, and trauma level; and model 3 was adjusted for age, race, body mass index, payment, hospital bed size, trauma level, systolic blood pressure (SBP), heart rate, respiratory rate, Glasgow Coma Scale score, temperature, pulse oximetry, respiratory assistance, alcohol use disorder, bleeding disorder, congestive heart failure, smoking, chronic kidney failure, hypertension, steroid use, cirrhosis, anticoagulant therapy, substance abuse disorder, and AIS scores of the liver, spleen, pancreas, head, neck, chest, spine, and upper and lower extremities.

#### Sensitivity Analysis

Inverse probability weighting was used as a sensitivity analysis to address potential selection bias by creating a pseudo-population in which baseline characteristics were balanced among the OS, SAE, and observation groups. We calculated propensity scores using logistic regression, incorporating all clinically relevant covariates (eAppendix in Supplement 1), applied stabilized weights to minimize variance, and assessed intergroup balance using standardized mean differences less than 0.1. Subsequently, the survey package was used to conduct weighted Cox proportional hazards regression, weighted logistic regression, and weighted linear regression analyses for the outcomes.

Subgroup analysis of patients presenting with hypotension (SBP <90 mm Hg) and normotension (SBP ≥90 mm Hg) was performed. In addition, outcomes of initial OS were compared with those in whom SAE and observation failed. The adjusted variables for subgroup analysis were detailed in the eAppendix in Supplement 1. All statistical analyses and graphical representations were performed using RStudio, version 4.4.1 (Posit PBC). Statistical significance was defined as a 2-tailed *P* < .05.

## Results

### Baseline Characteristics of Overall Cohort and Subgroups

**Figure 1.**
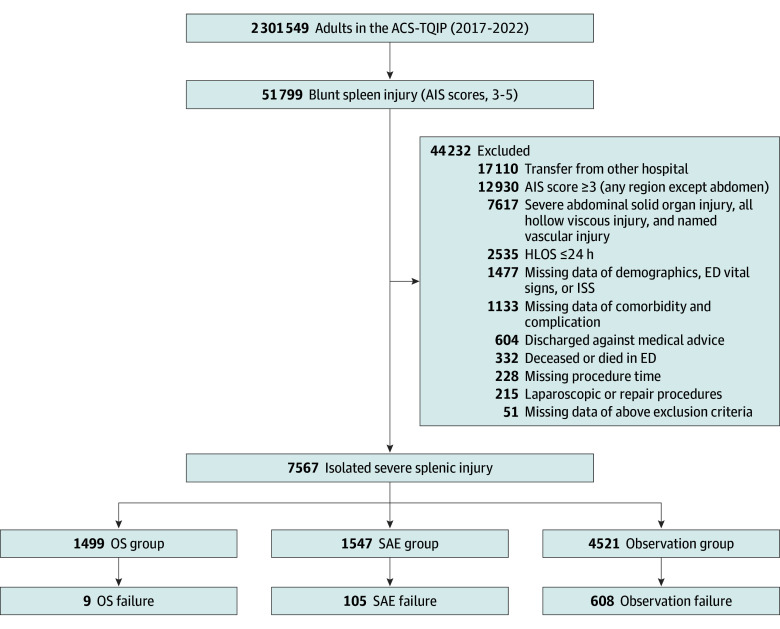
Patient Selection Flow Diagram ACS-TQIP indicates American College of Surgeons Trauma Quality Improvement Program; AIS, abbreviated injury scale; ED, emergency department; HLOS, hospital length of stay; ISS, Injury Severity Score; OS, operative splenectomy; SAE, splenic angioembolization.

A total of 7567 patients (median [IQR] age, 36 [25-55] years; 4901 male [64.8%] and 2666 [35.2%] female) with isolated severe blunt splenic injuries were included (Figure 1). Of these, 1499 patients (19.8%) were in the OS group, 1547 (20.4%) in the SAE group, and 4521 (59.7%) in the observation group. The overall demographic and clinical characteristics and outcomes of the study groups are given in eTables 1 and 2 in Supplement 1, respectively. The overall mortality rate was 1.2%, and the morbidity rate was 6.9%. In our main cohort, risk of acute kidney injury, acute respiratory distress syndrome, deep and organ space surgical site infection, unplanned intubation, and unplanned operations was higher in the operative group (eTable 2 in Supplement 1). Notably, risk of VAP was higher as well, with no difference in ventilator days between groups (eTable 2 in Supplement 1).

For subgroup analysis, the unadjusted baseline epidemiologic and clinical characteristics and outcomes of the hypotension subgroup are given in eTables 3 and 4 in Supplement 1, respectively. The unadjusted baseline epidemiologic and clinical characteristics and outcomes of the normotension subgroup are given in eTables 5 and 6 in Supplement 1, respectively. The unadjusted baseline characteristics and outcomes of the failure subgroup are given in eTables 7 and 8 in Supplement 1, respectively.

### Multivariable Analysis for Mortality

**Figure 2.**
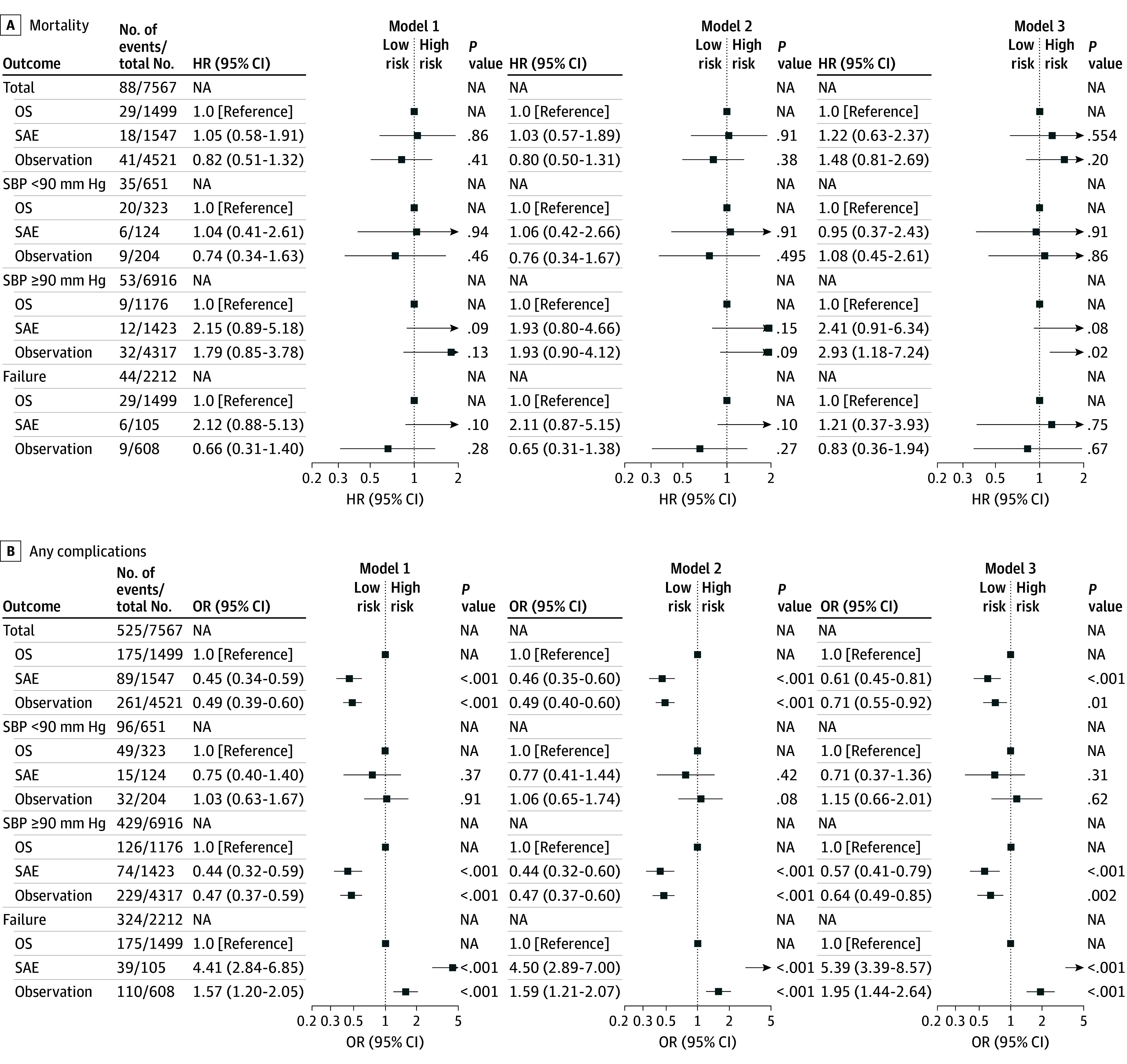
Multivariable Cox Proportional Hazards Regression Analysis for Mortality and Multivariable Logistic Regression Analysis for Any Complications HR indicates hazard ratio; NA, not applicable; OR, odds ratio; OS, operative splenectomy; SAE, splenic angioembolization; SBP, systolic blood pressure.

Results of multivariable Cox proportional hazards regression analysis for mortality are shown in Figure 2A. Compared with the OS group, there was no significant difference in mortality risk for the SAE group (HR, 1.22; 95% CI, 0.63-2.37; *P* = .55) or observation group (HR, 1.48; 95% CI, 0.81-2.69; *P* = .20) (Figure 2A). Among patients presenting with hypotension, there was also no significant mortality difference compared with the OS group (HR, 0.95; 95% CI, 0.37-2.43; *P* = .91 for SAE; HR, 1.08; 95% CI, 0.45-2.61; *P* = .86 for observation). In the subgroup in whom initial SAE or observation failed, there was similarly no significant difference in mortality risk compared with initial OS (HR, 1.21; 95% CI, 0.37-3.93; *P* = .75 for SAE; HR, 0.83; 95% CI, 0.36-1.94; *P* = .67 for observation).

### Multivariable Analysis for Complications

Multivariable logistic regression analysis for any complications is summarized in Figure 2B. The risk of any complication was significantly lower in the SAE (OR, 0.61; 95% CI, 0.45-0.81; *P* < .001) and observation (OR, 0.71; 95% CI, 0.55-0.92; *P* = .01) groups when compared with the OS group. This finding was similar in the normal SBP subgroup. In the hypotension subgroup, there were no significant differences in complication rate (OR, 0.71; 95% CI, 0.37-1.36; *P* = .30 for SAE; OR, 1.15; 95% CI, 0.66-2.01; *P* = .62 for observation). In the failure subgroup, the risk of any complication was significantly higher in those in whom SAE (OR, 5.39; 95% CI, 3.39-8.57; *P* < .001) and observation (OR, 1.95; 95% CI, 1.44-2.64; *P* < .001) failed.

The effect size of initial management failure and specific complications are given in Table 1. Regarding failure, the adjusted OR for SAE was 18.47 (95% CI, 8.90-38.34; *P* < .001), and the adjusted OR for observation was 64.75 (95% CI, 31.63-132.52; *P* < .001). The risk of VAP in the SAE and observation groups was significantly lower (OR, 0.09; 95% CI, 0.01-0.81; *P* = .03; OR, 0.10; 95% CI, 0.02-0.50; *P* = .005, respectively).

**Table 1.** Association of SAE or Observation vs OS With Failure and Complications Using Multivariable Logistic Regression and IPW

Outcome	Multivariable logistic regression			IPW	
	No. of events/total No.	OR (95% CI)	*P* value	OR (95% CI)	*P* value
Failure					
OS	8/1499	1.0 [Reference]	NA	1.0 [Reference]	NA
SAE	105/1547	18.47 (8.90-38.34)	<.001	18.43 (7.96-42.67)	<.001
Observation	608/4521	64.75 (31.63-132.52)	<.001	52.84 (23.32-119.71)	<.001
**Complications**					
AKI					
OS	24/1499	1.0 [Reference]	NA	1.0 [Reference]	NA
SAE	6/1547	0.43 (0.15-1.22)	.11	0.55 (0.21-1.44)	.22
Observation	29/4521	1.51 (0.71-3.20)	.28	1.39 (0.73-2.62)	.32
ARDS					
OS	6/1499	1.0 [Reference]	NA	1.0 [Reference]	NA
SAE	4/1547	0.74 (0.16-3.50)	.70	1.69 (0.43-6.54)	.45
Observation	2/4521	0.18 (0.03-1.18)	.07	0.16 (0.03-0.82)	.03
Cardiac arrest					
OS	19/1499	1.0 [Reference]	NA	1.0 [Reference]	NA
SAE	5/1547	0.47 (0.16-1.44)	.19	0.36 (0.09-1.37)	.13
Observation	18/4521	0.80 (0.33-1.95)	.62	0.64 (0.20-2.00)	.44
Unplanned intubation					
OS	32/1499	1.0 [Reference]	NA	1.0 [Reference]	NA
SAE	16/1547	0.58 (0.30-1.12)	.10	0.39 (0.18-0.84)	.02
Observation	44/4521	0.57 (0.32-1.03)	.06	0.52 (0.27-0.99)	.04
Unplanned visit to operating room					
OS	48/1499	1.0 [Reference]	NA	1.0 [Reference]	NA
SAE	22/1547	0.77 (0.44-1.35)	.36	0.46 (0.19-1.13)	.09
Observation	56/4521	1.03 (0.63-1.68)	.91	0.60 (0.26-1.38)	.23
VAP	18/7567				
OS		1.0 [Reference]	NA	1.0 [Reference]	NA
SAE	1/1547	0.09 (0.01-0.81)	.03	0.09 (0.01-0.72)	.02
Observation	3/4521	0.10 (0.02-0.50)	.005	0.13 (0.03-0.56)	.006
Alcohol withdrawal syndrome					
OS	19/1499	1.0 [Reference]	NA	1.0 [Reference]	NA
SAE	7/1547	0.47 (0.18-1.24)	.13	0.41 (0.15-1.10)	.08
Observation	24/4521	0.43 (0.20-0.92)	.03	0.49 (0.23-1.02)	.06
Pressure ulcer					
OS	12/1499	1.0 [Reference]	NA	1.0 [Reference]	NA
SAE	2/1547	0.20 (0.04-1.00)	.05	0.44 (0.07-2.71)	.38
Observation	8/4521	0.24 (0.08-0.78)	.02	0.34 (0.12-0.92)	.04

Abbreviations: AKI, acute kidney injury; ARDS, acute respiratory distress syndrome; IPW, inverse probability weighting; NA, not applicable; OR, operating room; OS, operative splenectomy; SAE, splenic angioembolization; VAP, ventilator associated pneumonia.

### Multivariable Analysis for Other Outcomes

Multivariable linear regression analysis of continuous variable outcomes is given in Table 2. SAE (β = −1.44; 95% CI, −1.79 to −1.09; *P* < .001) and observation (β = −1.41; 95% CI, −1.73 to −1.09; *P* < .001) showed a negative association with HLOS days. In addition, SAE (β = −0.74; 95% CI, −1.02 to −0.46; *P* < .001) and observation (β = −0.47; 95% CI, −0.73 to −0.20; *P* < .001) showed a negative association with ICULOS days. There was no significant difference among the 3 groups regarding ventilation days. The same results were observed in normal SBP subgroup. In the hypotension subgroup, SAE was also negatively associated with HLOS days (β = −1.65; 95% CI, −3.24 to −0.06; *P* = .04) and ICULOS days (β = −1.45; 95% CI, −2.62 to −0.29; *P* = .01). However, SAE (β = 2.50; 95% CI, 1.27-3.73; *P* < .001) and observation (β = 0.71; 95% CI, 0.07-1.35; *P* = .03) showed a positive association with HLOS days in the failure subgroup.

**Table 2.** SAE or Observation vs OS Outcomes Using Multivariable Linear Regression and IPW

Outcome	No.	Mean (SD)	Multivariable linear regression		IPW	
			β (95% CI)	*P* value	β (95% CI)	*P* value
**Total**						
HLOS days	7567	6.57 (5.27)	NA	NA	NA	NA
OS	1499	8.38 (6.53)	0 [Reference]	NA	0 [Reference]	NA
SAE	1547	6.35 (4.17)	−1.44 (−1.79 to −1.09)	<.001	−1.77 (−2.24 to −1.30)	<.001
Observation	4521	6.04 (5.00)	−1.41 (−1.73 to −1.09)	<.001	−1.60 (−2.05 to −1.14)	<.001
ICULOS days	5183	3.61 (3.47)	NA	NA	NA	NA
OS	1050	4.55 (4.96)	0 [Reference]	NA	0 [Reference]	NA
SAE	1244	3.28 (2.40)	−0.74 (−1.02 to −0.46)	<.001	−0.87 (−1.26 to −0.48)	<.001
Observation	2889	3.42 (3.13)	−0.47 (−0.73 to −0.20)	<.001	−0.49 (−0.89 to −0.09)	.02
Ventilation days	877	3.75 (4.75)	NA	NA	NA	NA
OS	480	3.55 (4.24)	0 [Reference]	NA	0 [Reference]	NA
SAE	111	3.46 (4.23)	−0.02 (−0.98 to 0.94)	.97	0.28 (−1.10 to 1.67)	.69
Observation	286	4.20 (5.65)	0.50 (−0.26 to 1.26)	.20	0.50 (−0.55 to 1.55)	.35
**SBP <90 mm Hg**						
HLOS days	651	8.83 (7.69)	NA	NA	NA	NA
OS	323	9.40 (8.31)	0 [Reference]	NA	0 [Reference]	NA
SAE	124	7.48 (4.80)	−1.65 (−3.24 to −0.06)	.04	−1.67 (−2.89 to −0.46)	.007
Observation	204	8.74 (8.01)	−0.13 (−1.58 to 1.32)	.86	−0.48 (−2.00 to 1.04)	.54
ICULOS days	549	4.78 (5.22)	NA	NA	NA	NA
OS	269	5.18 (6.05)	0 [Reference]	NA	0 [Reference]	NA
SAE	113	3.92 (2.77)	−1.45 (−2.62 to −0.29)	.01	−1.39 (−2.34 to −0.44)	.004
Observation	167	4.71 (4.99)	−0.49 (−1.59 to 0.61)	.38	−0.43 (−1.62 to 0.77)	.48
Ventilation days	205	3.66 (4.39)	NA	NA	NA	NA
OS	147	3.50 (3.75)	0 [Reference]	NA	0 [Reference]	NA
SAE	18	3.83 (3.55)	−0.08 (−2.30 to 2.15)	.95	0.03 (−1.77 to 1.82)	.98
Observation	40	4.20 (6.50)	0.69 (−1.05 to 2.43)	.44	0.38 (−1.30 to 2.06)	.66
**SBP ≥90 mm Hg**						
HLOS days	6916	6.36 (4.93)	NA	NA	NA	NA
OS	1176	8.10 (5.93)	0 [Reference]	NA	0 [Reference]	NA
SAE	1423	6.25 (4.10)	−1.44 (−1.80 to −1.09)	<.001	−1.80 (−2.31 to −1.28)	<.001
Observation	4317	5.91 (4.77)	−1.53 (−1.86 to −1.20)	<.001	−1.83 (−2.30 to −1.35)	<.001
ICULOS days	4634	3.47 (3.18)	NA	NA	NA	NA
OS	781	4.33 (4.51)	0 [Reference]	NA	0 [Reference]	NA
SAE	1131	3.21 (2.35)	−0.65 (−0.93 to −0.36)	<.001	−0.81 (−1.22 to −0.41)	<.001
Observation	2722	3.34 (2.96)	−0.45 (−0.72 to −0.19)	<.001	−0.55 (−0.95 to −0.14)	.009
Ventilation days	672	3.78 (4.86)	NA	NA	NA	NA
OS	333	3.57 (4.44)	0 [Reference]	NA	0 [Reference]	NA
SAE	93	3.39 (4.36)	0.14 (−0.96 to 1.23)	.80	−0.12 (−1.33 to 1.09)	.84
Observation	246	4.20 (5.52)	0.58 (−0.29 to 1.45)	.19	0.64 (−0.55 to 1.83)	.29
**Failure**						
HLOS days	2212	8.63 (6.65)	NA	NA	NA	NA
OS	1499	8.38 (6.53)	0 [Reference]	NA	0 [Reference]	NA
SAE	105	10.76 (6.07)	2.50 (1.27 to 3.73)	<.001	2.44 (1.14 to 3.75)	<.001
Observation	608	8.88 (6.96)	0.71 (0.07 to 1.35)	.03	1.20 (0.19 to 2.21)	.02
ICULOS days	1609	4.61 (4.84)	NA	NA	NA	NA
OS	1050	4.55 (4.96)	0 [Reference]	NA	0 [Reference]	NA
SAE	98	5.13 (4.24)	0.89 (−0.07 to 1.85)	.07	0.23 (−0.48 to 0.94)	.53
Observation	461	4.63 (4.68)	0.46 (−0.09 to 1.02)	.10	0.60 (−0.29 to 1.49)	.19
Ventilation days	589	3.72 (4.59)	NA	NA	NA	NA
OS	480	3.55 (4.24)	0 [Reference]	NA	0 [Reference]	NA
SAE	24	4.67 (6.30)	0.83 (−1.03 to 2.69)	.38	−0.45 (−1.70 to 0.80)	.48
Observation	85	4.46 (5.75)	0.65 (−0.45 to 1.76)	.24	0.00 (−1.08 to 1.09)	>.99

Abbreviations: HLOS, hospital length of stay; ICULOS, intensive care unit length of stay; IPW, inverse probability weighting; NA, not applicable; OS, operative splenectomy; SAE, splenic angioembolization.

### Sensitivity Analysis of Outcomes

**Figure 3.**
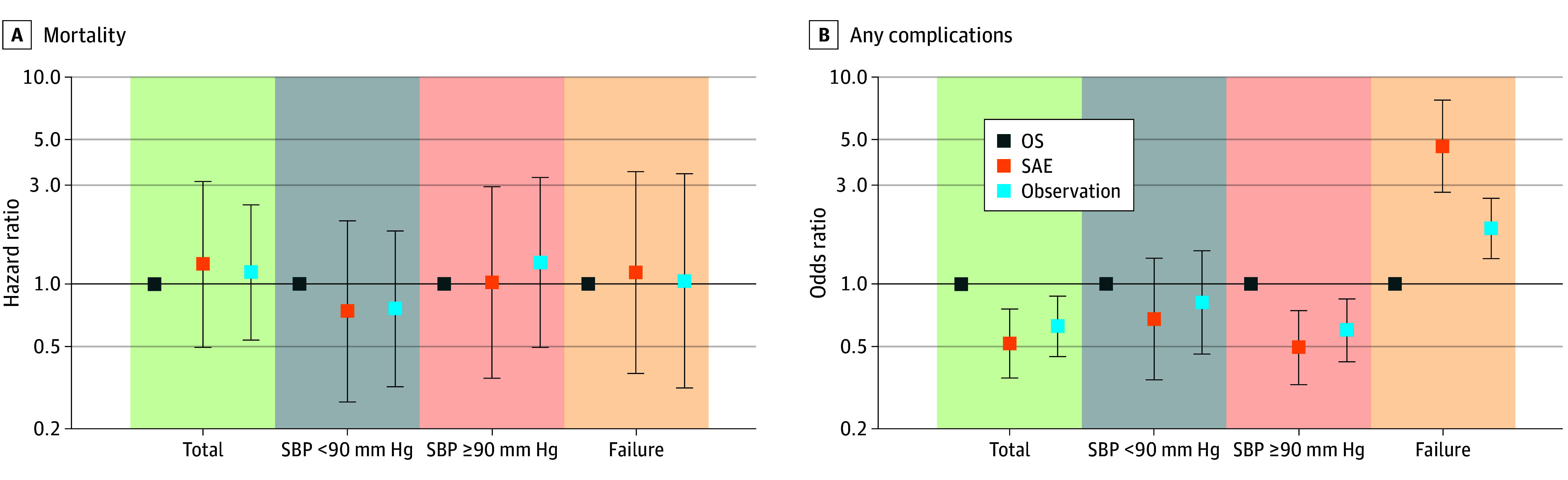
Inverse Probability Weighting Analysis for Mortality and Any Complications Horizontal line at 1.0 indicates reference; error bars, 95% CIs. OS indicates operative splenectomy; SAE, splenic angioembolization; SBP, systolic blood pressure.

Consistent associations were found among the 3 groups regarding mortality (HR, 1.25; 95% CI, 0.50-3.13; *P* = .64 for SAE; HR, 1.14; 95% CI, 0.54-2.41; *P* = .73 for observation) (Figure 3A). In subgroup analysis, there was still no significant difference in mortality risk (Figure 3A). Sensitivity analysis confirmed that risk of any complications was significantly lower in the SAE (OR, 0.52; 95% CI, 0.35-0.75; *P* < .001) and observation (OR, 0.63; 95% CI, 0.45-0.87; *P* = .006) groups (Figure 3B). In the failure subgroup, risk of any complications increased significantly in failure of SAE (OR: 4.62, 95%CI: 2.77-7.68, *P* < .001) and failure of observation (OR, 1.85; 95% CI, 1.32-2.59; *P* < .001) (Figure 3B).

Among specific complications, the risks of VAP in the SAE and observation groups and pressure ulcer in the observation group were confirmed (Table 1). Association of SAE or observation compared with OS with HLOS, ICULOS, and ventilation days also were confirmed (Table 2).

## Discussion

The mainstay of treatment for severe blunt splenic injury has become splenic salvage, by angioembolization or close observation, with splenectomy reserved for patients with the most unstable conditions or those with multiple injuries otherwise requiring emergency operative intervention. However, all management options are prone to well-documented risks, and guidelines regarding patient selection for initial management and regarding surveillance for management failure are still being refined.

Angioembolization is quickly becoming more accessible even in emergency settings, pushing the boundaries of feasible treatment options for patients with unstable conditions.^14,15^ Interventional radiology is being integrated into the management algorithm with new benchmarks for door-to-puncture times in trauma centers.^16,17^ Furthermore, improvements in prehospital and emergency department resuscitation will safely bridge more patients to nonoperative options, such as SAE. Therefore, continuing to hone our understanding of the risks of these treatment options is critical to improving patient outcomes and quality of care.

In this retrospective cohort study, most patients (59.7%) were initially managed with observations, which is like prior studies.^18,19^ As expected, patients with unstable vital signs were more likely to be managed with OS. Patients older than 55 years were less likely to undergo observation and were more often treated with initial operative or angiographic intervention. This approach aligns with the World Society of Emergency Surgery guidelines, which support a higher likelihood of nonoperative management failure in this older patient population, although age alone is not a contraindication.^1^ Although mortality was significantly higher in the OS group in univariable analysis, there was no difference in mortality based on initial management in the overall cohort or in any subgroup multivariable analysis. This finding is consistent with prior studies^9,20,21^ showing no meaningful mortality difference after accounting for severity of trauma and baseline characteristics. The overall mortality rate in this study was quite low, which may be a product of selection criteria in this group of isolated splenic injuries.

When compared with splenectomy, nonoperative management (SAE or observation) had a lower risk of complications. Although OS may lend itself to more definitive hemorrhage control, it carries risk of any maximally invasive procedure. These infectious and inflammatory postoperative complications may be attributable to changes in immunologic function after splenectomy in addition to common risks after open surgery. There have been mixed results from prior studies^6,10,14,18,20,22^ regarding morbidity after management strategy for blunt splenic injury. Operative management has been previously associated with higher morbidity, particularly infectious complications.^6,20^ However, other studies have shown no difference in complication rates or higher morbidity with SAE.^10,14,18,22^ Although retrospective, our study is, to our knowledge, the largest recent review of high-grade splenic injuries examining all 3 treatment modalities. SAE and observation also showed a benefit in decreased HLOS and ICULOS.

SAE is being increasingly used in patients with hemodynamic instability.^14,23^ In our subgroup analysis of patients who presented with hypotension, there were no differences in mortality or complications by management type. These findings are consistent with another recent ACS-TQIP study of hemodynamically unstable patients with severe blunt splenic injuries in which SAE and OS had similar complication and mortality rates.^15^ An investigation by Guinto et al,^14^ however, using the National Trauma Data Bank found no higher risk of mortality in patients with hypotension who underwent SAE over observation. However, they found that the failure rate of SAE was high and more organ-space infections occurred in patients undergoing SAE. Our study contributes to the growing literature that supports consideration of nonoperative management in hemodynamically unstable patients.

An important consideration when selecting nonoperative management for high-grade splenic injuries is the consequence of treatment failure. In our study, a subgroup analysis of patients in whom initial observation or SAE failed found that there was no increased risk of mortality but complications increased as did HLOS and ICULOS. This finding has important implications for clinical practice because some patients may experience more significant consequences from these complications than others. More investigation is also needed into patients with concomitant brain injuries, for example, who may not tolerate initial treatment failure.^24^ Patient selection, therefore, is critical to safe practice with these severe injuries. To our knowledge, this is the largest recent comparison of outcomes of the 3 primary management strategies for severe blunt splenic injury in an area that is undergoing rapid change in practice patterns.

### Limitations

This study has limitations inherent to its retrospective design and data source. Studies using large databases are subject to selection bias, missing granular information, and data inaccuracy, such as AIS score. Although we used multiple statistical methods to adjust for confounding factors, some information, such as lowest SBP and highest heart rate, is not available in the database. Due to the nature of the database, it was not possible to determine, for example, whether patients presenting with hypotension responded to resuscitation and stabilized before intervention (or observation), and diagnosis of splenic injury may sometimes be made intraoperatively (ie, after management decisions) in some hemodynamically unstable patients. Therefore, residual confounding factors cannot be ruled out.

In this study, we included only isolated splenic injury. A significant amount of splenic injury is associated with severe intra-abdominal or extra-abdominal injury, which may meaningfully impact the treatment approach. Therefore, management of splenic injury must always be performed in the context of whole patients with their individual injury patterns, and multiple traumas should be evaluated in future research. For the analysis of specific complications, the low event rates may affect the effectiveness of statistics. Laparoscopic or repair procedures were also not included in this study because patient numbers were too small and these procedures were often considered in hemodynamically stable patients.

## Conclusions

This retrospective cohort study of patients with isolated severe blunt splenic injury found that patients undergoing SAE and observation had fewer complications and shorter LOS compared with those receiving observation, with no difference in mortality. Furthermore, in patients with hypotension on admission, nonoperative management (either with SAE or observation) was associated with no increased risk of complications or mortality and shorter LOS. In patients who in whom observation or SAE failed, there was a higher risk of complications and longer LOS without an increased mortality risk. These findings add strong support to the increasing literature showing the safety of nonoperative management for even high-grade splenic injury and the feasibility of using SAE in hemodynamically unstable patients. With careful patient selection, nonoperative management and splenic salvage may be possible and preferred even in severely injured patients.

## Notes

Supplement 1. **eAppendix.** Adjusted Variables **eTable 1.** The Baseline of OS, SAE, and Observation Groups **eTable 2.** Outcomes of OS, SAE, and Observation Groups **eTable 3.** The Baseline of OS, SAE, and Observation Groups (SBP <90 mm Hg) **eTable 4.** Outcomes of OS, SAE, and Observation Groups (SBP <90 mm Hg) **eTable 5.** The Baseline of OS, SAE, and Observation Groups (SBP ≥90 mm Hg) **eTable 6.** Outcomes of OS, SAE, and Observation Groups (SBP ≥90 mm Hg) **eTable 7.** The Baseline of OS, Failure of SAE, and Failure of Observation Groups **eTable 8.** Outcomes of OS, Failure of SAE, and Failure of Observation Groups

Supplement 2.
Data Sharing Statement
