# Supplementary material for: Outcomes of Isolated Severe Blunt Splenic Injury
Source: JAMA Netw Open. 2025 Sep 23;8(9):e2533266. doi: 10.1001/jamanetworkopen.2025.33266 (PMC12457976; doi:10.1001/jamanetworkopen.2025.33266)
Supplement: Supplement 1. — eAppendix. Adjusted Variables eTable 1. The Baseline of OS, SAE, and Observation Groups eTable 2. Outcomes of OS, SAE, and Observation Groups eTable 3. The Baseline of OS, SAE, and Observation Groups (SBP <90 mm Hg) eTable 4. Outcomes of OS, SAE, and Observation Groups (SBP <90 mm Hg) eTable 5. The Baseline of OS, SAE, and Observation Groups (SBP ≥90 mm Hg) eTable 6. Outcomes of OS, SAE, and Observation Groups (SBP ≥90 mm Hg) eTable 7. The Baseline of OS, Failure of SAE, and Failure of Observation Groups eTable 8. Outcomes of OS, Failure of SAE, and Failure of Observation Groups [file jamanetwopen-e2533266-s001.pdf]

Supplemental Online Content

Huang W, Braschi C, Jin F, Lewis M, Demetriades D. Outcomes of isolated severe blunt splenic injury. *JAMA Netw Open*. 2025;8(5):e2533266. doi:10.1001/jamanetworkopen.2025.33266

eAppendix. Adjusted Variables
eTable 1. The Baseline of OS, SAE, and Observation Groups
eTable 2. Outcomes of OS, SAE, and Observation Groups
eTable 3. The Baseline of OS, SAE, and Observation Groups (SBP <90 mm Hg)
eTable 4. Outcomes of OS, SAE, and Observation Groups (SBP <90 mm Hg)
eTable 5. The Baseline of OS, SAE, and Observation Groups (SBP ≥90 mm Hg)
eTable 6. Outcomes of OS, SAE, and Observation Groups (SBP ≥90 mm Hg)
eTable 7. The Baseline of OS, Failure of SAE, and Failure of Observation Groups
eTable 8. Outcomes of OS, Failure of SAE, and Failure of Observation Groups

This supplemental material has been provided by the authors to give readers additional information about their work.

© 2025 Huang W et al. *JAMA Network Open.*

eAppendix. Adjusted Variables

1, Total

Model 1: age
Model 2: age, race, BMI, payment, bed size, trauma level
Model 3: age, race, BMI, payment, bed size, trauma level, SBP, HR, RR, GCS, temperature, pulse oximetry, respiratory assistance, alcohol use disorder, bleeding disorder, congestive heart failure, smoking, chronic renal failure, hypertension, steroid use, cirrhosis, anticoagulant therapy, substance abuse disorder, AIS of liver, spleen, pancreas, head, neck, chest, spine, upper extremity, lower extremity.

2, SBP < 90 mmHg

Model 1: sex
Model 2: sex, SBP, HR
Model 3: sex, SBP, HR, respiratory assistance, smoking, chronic renal failure, cirrhosis, anticoagulant therapy, AIS of spleen, pancreas, and upper extremity

3, SBP ⩾ 90 mmHg

Model 1: age, sex
Model 2: age, sex, race, BMI, bed size, trauma level
Model 3: age, sex, race, BMI, bed size, trauma level, SBP, HR, RR, GCS, temperature, pulse oximetry, respiratory assistance, alcohol use disorder, bleeding disorder, congestive heart failure, smoking, chronic renal failure, steroid use, dementia, myocardial infarction, substance abuse disorder, AIS of liver, spleen, pancreas, head, spine, upper extremity, lower extremity.

4, Failure

Model 1: age
Model 2: age, bed size
Model 3: age, bed size, SBP, HR, RR, GCS, temperature, pulse oximetry, chronic renal failure, diabetes mellitus, COPD, mechanism, AIS of liver, spleen, kidney, pancreas, spine

© 2025 Huang W et al. *JAMA Network Open.*

eTable 1: the baseline of OS, SAE, and Observation groups

Variables	Total (n = 7567)	OS (n = 1499)	SAE (n = 1547)	Observation (n = 4521)	p
Age, Median (IQR)	36 (25, 55)	39 (28, 56)	38 (26, 56)	35 (24, 54)	< 0.001
≥55, n (%)	1896 (25.1)	403 (26.9)	417 (27.0)	1076 (23.8)	0.009
Sex, n (%)					
Male	4901 (64.8)	970 (64.7)	1026 (66.3)	2905 (64.3)	0.340
Female	2666 (35.2)	529 (35.3)	521 (33.7)	1616 (35.7)	
Race, n (%)					
Black	684 (9.0)	115 (7.7)	126 (8.1)	443 (9.8)	0.026
White	5978 (79.0)	1218 (81.3)	1245 (80.5)	3515 (77.7)	
Other[a]	785 (10.4)	141 (9.4)	148 (9.6)	496 (11.0)	
Unknow	120 (1.6)	25 (1.7)	28 (1.8)	67 (1.5)	
BMI, n (%)					
<30	5116 (67.6)	1056 (70.4)	1047 (67.7)	3013 (66.6)	0.037
≥30	1974 (26.1)	344 (22.9)	408 (26.4)	1222 (27.0)	
Unknow	477 (6.3)	99 (6.6)	92 (5.9)	286 (6.3)	
Hospital type, n (%)					
Non-Profit	6569 (86.8)	1323 (88.3)	1324 (85.6)	3922 (86.8)	0.348
Profit	945 (12.5)	169 (11.3)	210 (13.6)	566 (12.5)	
Government	51 (0.7)	7 (0.5)	13 (0.8)	31 (0.7)	
Unknow	2 (0.0)	0 (0.0)	0 (0.0)	2 (0.0)	
Payment, n (%)					
Insurance	3628 (47.9)	667 (44.5)	769 (49.7)	2192 (48.5)	0.075
Medicaid	1494 (19.7)	328 (21.9)	289 (18.7)	877 (19.4)	
Self-Pay	942 (12.4)	203 (13.5)	193 (12.5)	546 (12.1)	
Medicare	896 (11.8)	195 (13.0)	177 (11.4)	524 (11.6)	
Other	506 (6.7)	87 (5.8)	101 (6.5)	318 (7.0)	
Unknow	101 (1.3)	19 (1.3)	18 (1.2)	64 (1.4)	
Bed size, n (%)					
<200	421 (5.6)	73 (4.9)	94 (6.1)	254 (5.6)	0.087
201 to 400	2341 (30.9)	476 (31.8)	465 (30.1)	1400 (31.0)	
401 to 600	2184 (28.9)	453 (30.2)	411 (26.6)	1320 (29.2)	
More than 600	2621 (34.6)	497 (33.2)	577 (37.3)	1547 (34.2)	
Trauma Level, n (%)					
I	3420 (45.2)	711 (47.4)	707 (45.7)	2002 (44.3)	0.005
II	2479 (32.8)	429 (28.6)	499 (32.3)	1551 (34.3)	
III	24 (0.3)	7 (0.5)	3 (0.2)	14 (0.3)	
Unknow	1644 (21.7)	352 (23.5)	338 (21.8)	954 (21.1)	
ED first vital signs					
SBP, Median (IQR)	122 (107, 138)	110 (92, 127)	122 (107, 137)	127 (112, 141)	< 0.001
<90, n (%)	651 (8.6)	323 (21.5)	124 (8.0)	204 (4.5)	< 0.001
HR, Median (IQR)	90 (77, 104)	93 (80, 112)	88 (76, 102)	89 (77, 102)	< 0.001

© 2025 Huang W et al. *JAMA Network Open.*

⩾120, n (%)	695 (9.2)	248 (16.5)	103 (6.7)	344 (7.6)	< 0.001
RR, Median (IQR)	18 (16, 20)	19 (17, 22)	18 (16, 20)	18 (16, 20)	< 0.001
GCS, Median (IQR)	15 (15, 15)	15 (15, 15)	15 (15, 15)	15 (15, 15)	< 0.001
Temperature, n (%)					
<36℃	510 (6.7)	171 (11.4)	95 (6.1)	244 (5.4)	< 0.001
⩾36℃	6523 (86.2)	1170 (78.1)	1337 (86.4)	4016 (88.8)	
Unknow	534 (7.1)	158 (10.5)	115 (7.4)	261 (5.8)	
Pulse oximetry, n (%)					
<90%	133 (1.8)	42 (2.8)	15 (1.0)	76 (1.7)	< 0.001
⩾90%	7357 (97.2)	1435 (95.7)	1511 (97.7)	4411 (97.6)	
Unknow	77 (1.0)	22 (1.5)	21 (1.4)	34 (0.8)	
Respiratory assistance, n (%)					
Yes	135 (1.8)	35 (2.3)	20 (1.3)	80 (1.8)	0.022
No	7303 (96.5)	1441 (96.1)	1511 (97.7)	4351 (96.2)	
Unknow	129 (1.7)	23 (1.5)	16 (1.0)	90 (2.0)	
ISS, Median (IQR)	16 (10, 21)	21 (16, 26)	17 (14, 24)	14 (10, 17)	< 0.001
⩾25, n (%)	1198 (15.8)	614 (41.0)	305 (19.7)	279 (6.2)	< 0.001
Comorbidities, n (%)					
Alcohol Use Disorder	500 (6.6)	131 (8.7)	99 (6.4)	270 (6.0)	< 0.001
Bleeding Disorder	61 (0.8)	21 (1.4)	16 (1.0)	24 (0.5)	0.003
Chemotherapy for Cancer	30 (0.4)	6 (0.4)	6 (0.4)	18 (0.4)	0.998
Congestive Heart Failure	157 (2.1)	39 (2.6)	23 (1.5)	95 (2.1)	0.096
Smoking	2233 (29.5)	506 (33.8)	458 (29.6)	1269 (28.1)	< 0.001
Chronic Renal Failure	68 (0.9)	8 (0.5)	12 (0.8)	48 (1.1)	0.146
Cerebrovascular Accident	71 (0.9)	16 (1.1)	13 (0.8)	42 (0.9)	0.805
Diabetes Mellitus	579 (7.7)	106 (7.1)	123 (8.0)	350 (7.7)	0.618
Hypertension	1438 (19.0)	296 (19.7)	320 (20.7)	822 (18.2)	0.068
COPD	322 (4.3)	70 (4.7)	70 (4.5)	182 (4.0)	0.474
Steroid Use	40 (0.5)	7 (0.5)	4 (0.3)	29 (0.6)	0.187
Cirrhosis	165 (2.2)	49 (3.3)	39 (2.5)	77 (1.7)	< 0.001
Dementia	63 (0.8)	10 (0.7)	12 (0.8)	41 (0.9)	0.651
Anticoagulant Therapy	356 (4.7)	87 (5.8)	79 (5.1)	190 (4.2)	0.028
Angina Pectoris	3 (0.0)	1 (0.1)	0 (0.0)	2 (0.0)	0.551
Mental disorder	850 (11.2)	171 (11.4)	182 (11.8)	497 (11.0)	0.689
Myocardial Infarction	24 (0.3)	5 (0.3)	2 (0.1)	17 (0.4)	0.349
Peripheral Arterial Disease	32 (0.4)	10 (0.7)	6 (0.4)	16 (0.4)	0.262
Substance Abuse Disorder	1020 (13.5)	268 (17.9)	224 (14.5)	528 (11.7)	< 0.001
Any comorbidities	4287 (56.7)	950 (63.4)	903 (58.4)	2434 (53.8)	< 0.001
Mechanism, n (%)					
MVT	4488 (59.3)	859 (57.3)	937 (60.6)	2692 (59.5)	0.453
Fall	1704 (22.5)	354 (23.6)	340 (22.0)	1010 (22.3)	
Other	1375 (18.2)	286 (19.1)	270 (17.5)	819 (18.1)	
Abdominal solid organ AIS, n (%)					
Liver AIS=2	524 (6.9)	123 (8.2)	112 (7.2)	289 (6.4)	0.049
Spleen					

© 2025 Huang W et al. *JAMA Network Open.*

AIS=3	4271 (56.4)	372 (24.8)	574 (37.1)	3325 (73.5)	< 0.001
AIS=4	2098 (27.7)	513 (34.2)	668 (43.2)	917 (20.3)	
AIS=5	1198 (15.8)	614 (41.0)	305 (19.7)	279 (6.2)	
Kidney AIS=2	357 (4.7)	72 (4.8)	66 (4.3)	219 (4.8)	0.642
Pancreas AIS=2	93 (1.2)	49 (3.3)	11 (0.7)	33 (0.7)	< 0.001
AIS of each region, Median (IQR)					
Head	0 (0, 0)	0 (0, 0)	0 (0, 0)	0 (0, 0)	0.025
Face	0 (0, 1)	0 (0, 1)	0 (0, 1)	0 (0, 1)	0.779
Neck	0 (0, 0)	0 (0, 0)	0 (0, 0)	0 (0, 0)	0.138
Chest	0 (0, 2)	0 (0, 2)	0 (0, 2)	0 (0, 2)	0.188
Spine	0 (0, 0)	0 (0, 0)	0 (0, 0)	0 (0, 0)	< 0.001
Upper extremity	0 (0, 1)	0 (0, 1)	0 (0, 1)	0 (0, 1)	< 0.001
Lower extremity	0 (0, 1)	0 (0, 1)	0 (0, 1)	0 (0, 1)	< 0.001

BMI, Body Mass Index; SBP, systolic blood pressure; HR, heart rate; RR, respiratory rate; GCS, Glasgow Coma Scale; ISS, injury severity score; AIS, abbreviated injury score; ARDS, acute respiratory distress syndrome; [a] Any races which were not classified as White, Black, or Unknow were classified as other.

© 2025 Huang W et al. *JAMA Network Open.*

eTable 2: Outcomes of OS, SAE, and Observation groups

Variables	Total (n = 7567)	OS (n = 1499)	SAE (n = 1547)	Observation (n = 4521)	p
Mortality, n (%)	88 (1.2)	29 (1.9)	18 (1.2)	41 (0.9)	0.006
Failure, n (%)	721 (9.5)	8 (0.5)	105 (6.8)	608 (13.4)	< 0.001
HLOS days, Median (IQR)	5 (4, 7)	6 (5, 9)	5 (4, 7)	5 (3, 7)	< 0.001
ICULOS days, Median (IQR)	3 (2, 4)	3 (2, 5)	3 (2, 4)	3 (2, 4)	< 0.001
Ventilation days, Median (IQR)	2 (2, 4)	2 (2, 3)	2 (2, 4)	2 (2, 4)	0.356
Complications, n (%)					
AKI	59 (0.8)	24 (1.6)	6 (0.4)	29 (0.6)	< 0.001
ARDS	12 (0.2)	6 (0.4)	4 (0.3)	2 (0.0)	0.004
Cardiac Arrest	42 (0.6)	19 (1.3)	5 (0.3)	18 (0.4)	< 0.001
Deep SSI	6 (0.1)	4 (0.3)	0 (0.0)	2 (0.0)	0.026
Organ Space SSI	8 (0.1)	7 (0.5)	0 (0.0)	1 (0.0)	< 0.001
Superficial SSI	8 (0.1)	4 (0.3)	1 (0.1)	3 (0.1)	0.117
Severe Sepsis	35 (0.5)	10 (0.7)	5 (0.3)	20 (0.4)	0.358
VTE	65 (0.9)	20 (1.3)	14 (0.9)	31 (0.7)	0.061
DVT	44 (0.6)	13 (0.9)	6 (0.4)	25 (0.6)	0.203
PE	32 (0.4)	10 (0.7)	9 (0.6)	13 (0.3)	0.081
Myocardial Infarction	11 (0.1)	4 (0.3)	2 (0.1)	5 (0.1)	0.361
Stroke	15 (0.2)	2 (0.1)	3 (0.2)	10 (0.2)	0.938
Unplanned Intubation	92 (1.2)	32 (2.1)	16 (1.0)	44 (1.0)	0.001
Unplanned Admission to ICU	195 (2.6)	47 (3.1)	31 (2.0)	117 (2.6)	0.143
Unplanned Visit to OR	126 (1.7)	48 (3.2)	22 (1.4)	56 (1.2)	< 0.001
CAUTI	16 (0.2)	3 (0.2)	3 (0.2)	10 (0.2)	1.000
CLABI	0 (0.0)	0 (0.0)	0 (0.0)	0 (0.0)	1.000
VAP	18 (0.2)	14 (0.9)	1 (0.1)	3 (0.1)	< 0.001
Alcohol Withdrawal Syndrome	50 (0.7)	19 (1.3)	7 (0.5)	24 (0.5)	0.005
Pressure Ulcer	22 (0.3)	12 (0.8)	2 (0.1)	8 (0.2)	0.001
Any Complications	525 (6.9)	175 (11.7)	89 (5.8)	261 (5.8)	< 0.001

HLOS, hospital length of stay; ICULOS, intensive care unit length of stay; AKI, acute kidney injury; ARDS, acute respiratory distress syndrome; SSI, surgical site infection; VTE: venous thromboembolism; DVT, deep vein thrombosis; PE, pulmonary embolism; VAP, ventilator associated pneumonia; CAUTI: catheter associated urinary tract infection; CLABI: central line associated bloodstream infection; OR, operating room.

© 2025 Huang W et al. *JAMA Network Open.*

eTable 3: the baseline of OS, SAE, and Observation groups (SBP <90 mm Hg)

Variables	Total (n = 651)	OS (n = 323)	SAE (n = 124)	Observation (n = 204)	p
Age, Median (IQR)	51 (35, 64)	49 (33, 63)	51 (36, 66)	53 (36, 64)	0.553
≥55, n (%)	281 (43.2)	130 (40.2)	59 (47.6)	92 (45.1)	0.299
Sex, n (%)					
Male	387 (59.4)	177 (54.8)	86 (69.4)	124 (60.8)	0.017
Female	264 (40.6)	146 (45.2)	38 (30.6)	80 (39.2)	
Race, n (%)					
Black	49 (7.5)	20 (6.2)	11 (8.9)	18 (8.8)	0.745
White	541 (83.1)	273 (84.5)	103 (83.1)	165 (80.9)	
Other[a]	51 (7.8)	24 (7.4)	8 (6.5)	19 (9.3)	
Unknow	10 (1.5)	6 (1.9)	2 (1.6)	2 (1.0)	
BMI, n (%)					
<30	422 (64.8)	218 (67.5)	81 (65.3)	123 (60.3)	0.287
≥30	185 (28.4)	84 (26.0)	38 (30.6)	63 (30.9)	
Unknow	44 (6.8)	21 (6.5)	5 (4.0)	18 (8.8)	
Hospital type, n (%)					
Non-Profit	576 (88.5)	284 (87.9)	108 (87.1)	184 (90.2)	0.525
Profit	70 (10.8)	37 (11.5)	16 (12.9)	17 (8.3)	
Government	4 (0.6)	2 (0.6)	0 (0.0)	2 (1.0)	
Unknow	1 (0.2)	0 (0.0)	0 (0.0)	1 (0.5)	
Payment, n (%)					
Insurance	233 (35.8)	117 (36.2)	45 (36.3)	71 (34.8)	0.927
Medicaid	146 (22.4)	76 (23.5)	22 (17.7)	48 (23.5)	
Self-Pay	72 (11.1)	34 (10.5)	15 (12.1)	23 (11.3)	
Medicare	154 (23.7)	76 (23.5)	29 (23.4)	49 (24.0)	
Other	35 (5.4)	15 (4.6)	10 (8.1)	10 (4.9)	
Unknow	11 (1.7)	5 (1.5)	3 (2.4)	3 (1.5)	
Bed size, n (%)					
<200	39 (6.0)	13 (4.0)	10 (8.1)	16 (7.8)	0.442
201 to 400	201 (30.9)	100 (31.0)	42 (33.9)	59 (28.9)	
401 to 600	192 (29.5)	97 (30.0)	32 (25.8)	63 (30.9)	
More than 600	219 (33.6)	113 (35.0)	40 (32.3)	66 (32.4)	
Trauma Level, n (%)					
I	307 (47.2)	154 (47.7)	55 (44.4)	98 (48.0)	0.901
II	210 (32.3)	98 (30.3)	43 (34.7)	69 (33.8)	
III	5 (0.8)	3 (0.9)	1 (0.8)	1 (0.5)	
Unknow	129 (19.8)	68 (21.1)	25 (20.2)	36 (17.6)	
ED first vital signs					
SBP, Median (IQR)	80 (72, 85)	79 (71, 84)	80 (73, 85)	82 (74, 86)	0.010
HR, Median (IQR)	91 (77, 108)	93 (80, 112)	90 (74, 105)	90 (76, 103)	0.022
≥120, n (%)	95 (14.6)	57 (17.6)	14 (11.3)	24 (11.8)	0.090
RR, Median (IQR)	18 (16, 22)	19 (16, 22)	19 (16, 22)	18 (16, 21)	0.551
GCS, Median (IQR)	15 (15, 15)	15 (15, 15)	15 (15, 15)	15 (15, 15)	0.230

© 2025 Huang W et al. *JAMA Network Open.*

Temperature, n (%)					
<36℃	101 (15.5)	58 (18.0)	14 (11.3)	29 (14.2)	0.264
≥36℃	455 (69.9)	214 (66.3)	91 (73.4)	150 (73.5)	
Unknow	95 (14.6)	51 (15.8)	19 (15.3)	25 (12.3)	
Pulse oximetry, n (%)					
<90%	32 (4.9)	19 (5.9)	4 (3.2)	9 (4.4)	0.844
≥90%	601 (92.3)	295 (91.3)	117 (94.4)	189 (92.6)	
Unknow	18 (2.8)	9 (2.8)	3 (2.4)	6 (2.9)	
Respiratory assistance, n (%)					
Yes	25 (3.8)	13 (4.0)	2 (1.6)	10 (4.9)	0.083
No	607 (93.2)	299 (92.6)	122 (98.4)	186 (91.2)	
Unknow	19 (2.9)	11 (3.4)	0 (0.0)	8 (3.9)	
ISS, Median (IQR)	17 (11, 25)	20 (16, 26)	17 (14, 25)	14 (10, 17)	< 0.001
≥25, n (%)	183 (28.1)	134 (41.5)	32 (25.8)	17 (8.3)	< 0.001
Comorbidities, n (%)					
Alcohol Use Disorder	72 (11.1)	35 (10.8)	16 (12.9)	21 (10.3)	0.753
Bleeding Disorder	15 (2.3)	10 (3.1)	3 (2.4)	2 (1.0)	0.296
Chemotherapy for Cancer	7 (1.1)	2 (0.6)	1 (0.8)	4 (2.0)	0.343
Congestive Heart Failure	29 (4.5)	13 (4.0)	5 (4.0)	11 (5.4)	0.736
Smoking	191 (29.3)	107 (33.1)	31 (25.0)	53 (26.0)	0.107
Chronic Renal Failure	10 (1.5)	3 (0.9)	1 (0.8)	6 (2.9)	0.155
Cerebrovascular Accident	22 (3.4)	8 (2.5)	4 (3.2)	10 (4.9)	0.333
Diabetes Mellitus	79 (12.1)	32 (9.9)	19 (15.3)	28 (13.7)	0.205
Hypertension	187 (28.7)	88 (27.2)	41 (33.1)	58 (28.4)	0.474
COPD	59 (9.1)	24 (7.4)	14 (11.3)	21 (10.3)	0.339
Steroid Use	7 (1.1)	4 (1.2)	0 (0.0)	3 (1.5)	0.553
Cirrhosis	37 (5.7)	22 (6.8)	9 (7.3)	6 (2.9)	0.122
Dementia	11 (1.7)	5 (1.5)	4 (3.2)	2 (1.0)	0.364
Anticoagulant Therapy	65 (10.0)	28 (8.7)	18 (14.5)	19 (9.3)	0.169
Angina Pectoris	1 (0.2)	0 (0.0)	0 (0.0)	1 (0.5)	0.491
Mental disorder	94 (14.4)	43 (13.3)	17 (13.7)	34 (16.7)	0.548
Myocardial Infarction	5 (0.8)	3 (0.9)	1 (0.8)	1 (0.5)	1.000
Peripheral Arterial Disease	12 (1.8)	8 (2.5)	0 (0.0)	4 (2.0)	0.249
Substance Abuse Disorder	108 (16.6)	58 (18.0)	23 (18.5)	27 (13.2)	0.295
Any comorbidities	457 (70.2)	225 (69.7)	93 (75.0)	139 (68.1)	0.401
Mechanism, n (%)					
MVT	273 (41.9)	143 (44.3)	42 (33.9)	88 (43.1)	0.353
Fall	265 (40.7)	124 (38.4)	58 (46.8)	83 (40.7)	
Other	113 (17.4)	56 (17.3)	24 (19.4)	33 (16.2)	
Abdominal solid organ AIS, n (%)					
Liver AIS=2	57 (8.8)	29 (9.0)	11 (8.9)	17 (8.3)	0.967
Spleen					
AIS=3	263 (40.4)	84 (26.0)	41 (33.1)	138 (67.6)	< 0.001
AIS=4	205 (31.5)	105 (32.5)	51 (41.1)	49 (24.0)	
AIS=5	183 (28.1)	134 (41.5)	32 (25.8)	17 (8.3)	
Kidney AIS=2	18 (2.8)	7 (2.2)	6 (4.8)	5 (2.5)	0.289

© 2025 Huang W et al. *JAMA Network Open.*

Pancreas AIS=2	7 (1.1)	7 (2.2)	0 (0.0)	0 (0.0)	0.038
AIS of each region, Median (IQR)					
Head	0 (0, 0)	0 (0, 0)	0 (0, 0)	0 (0, 0)	0.444
Face	0 (0, 0)	0 (0, 0)	0 (0, 0)	0 (0, 0)	0.902
Neck	0 (0, 0)	0 (0, 0)	0 (0, 0)	0 (0, 0)	0.360
Chest	0 (0, 1)	0 (0, 1)	0 (0, 1)	0 (0, 1)	0.553
Spine	0 (0, 0)	0 (0, 0)	0 (0, 0)	0 (0, 0)	0.651
Upper extremity	0 (0, 0.5)	0 (0, 0)	0 (0, 1)	0 (0, 1)	0.191
Lower extremity	0 (0, 0)	0 (0, 0)	0 (0, 0)	0 (0, 1)	0.374
Blood transfusion (4 hours), ml, mean (SD)					
Pack red blood cell	684 (844)	1015 (968)	516 (566)	265 (487)	< 0.001
Plasma	423 (614)	615 (679)	233 (392)	188 (477)	< 0.001

BMI, Body Mass Index; SBP, systolic blood pressure; HR, heart rate; RR, respiratory rate; GCS, Glasgow Coma Scale; ISS, injury severity score; AIS, abbreviated injury score; ARDS, acute respiratory distress syndrome; [a] Any races which were not classified as White, Black, or Unknow were classified as other.

© 2025 Huang W et al. *JAMA Network Open.*

eTable 4: outcomes of OS, SAE, and Observation groups (SBP <90 mm Hg)

Variables	Total (n = 651)	OS (n = 323)	SAE (n = 124)	Observation (n = 204)	p
Mortality, n (%)	35 (5.4)	20 (6.2)	6 (4.8)	9 (4.4)	0.649
Failure, n (%)	63 (9.7)	4 (1.2)	16 (12.9)	43 (21.1)	< 0.001
HLOS days, Median (IQR)	6 (5, 10)	7 (5, 10)	6 (4, 10)	6 (5, 10)	0.003
ICULOS days, Median (IQR)	3 (2, 5)	3 (2, 6)	3 (2, 4)	3 (2, 5)	0.873
Ventilation days, Median (IQR)	2 (2, 4)	2 (2, 3.5)	2.5 (2, 4)	2 (2, 4)	0.777
Complications, n (%)					
AKI	26 (4.0)	16 (5.0)	2 (1.6)	8 (3.9)	0.300
ARDS	5 (0.8)	3 (0.9)	1 (0.8)	1 (0.5)	1.000
Cardiac Arrest	16 (2.5)	10 (3.1)	2 (1.6)	4 (2.0)	0.658
Deep SSI	2 (0.3)	2 (0.6)	0 (0.0)	0 (0.0)	0.691
Organ Space SSI	1 (0.2)	1 (0.3)	0 (0.0)	0 (0.0)	1.000
Superficial SSI	0 (0.0)	0 (0.0)	0 (0.0)	0 (0.0)	1.000
Severe Sepsis	16 (2.5)	8 (2.5)	2 (1.6)	6 (2.9)	0.785
VTE	11 (1.7)	4 (1.2)	0 (0.0)	7 (3.4)	0.055
DVT	10 (1.5)	3 (0.9)	0 (0.0)	7 (3.4)	0.032
PE	1 (0.2)	1 (0.3)	0 (0.0)	0 (0.0)	1.000
Myocardial Infarction	3 (0.5)	2 (0.6)	0 (0.0)	1 (0.5)	1.000
Stroke	1 (0.2)	0 (0.0)	1 (0.8)	0 (0.0)	0.190
Unplanned Intubation	15 (2.3)	7 (2.2)	1 (0.8)	7 (3.4)	0.326
Unplanned Admission to ICU	28 (4.3)	11 (3.4)	5 (4.0)	12 (5.9)	0.389
Unplanned Visit to OR	26 (4.0)	15 (4.6)	5 (4.0)	6 (2.9)	0.646
CAUTI	4 (0.6)	2 (0.6)	1 (0.8)	1 (0.5)	1.000
CLABI	0 (0.0)	0 (0.0)	0 (0.0)	0 (0.0)	1.000
VAP	3 (0.5)	2 (0.6)	0 (0.0)	1 (0.5)	1.000
Alcohol Withdrawal Syndrome	9 (1.4)	5 (1.5)	1 (0.8)	3 (1.5)	1.000
Pressure Ulcer	5 (0.8)	4 (1.2)	0 (0.0)	1 (0.5)	0.582
Any Complications	96 (14.7)	49 (15.2)	15 (12.1)	32 (15.7)	0.643

HLOS, hospital length of stay; ICULOS, intensive care unit length of stay; AKI, acute kidney injury; ARDS, acute respiratory distress syndrome; SSI, surgical site infection; VTE: venous thromboembolism; DVT, deep vein thrombosis; PE, pulmonary embolism; VAP, ventilator associated pneumonia; CAUTI: catheter associated urinary tract infection; CLABI: central line associated bloodstream infection; OR, operating room.

© 2025 Huang W et al. *JAMA Network Open.*

eTable 5: the baseline of OS, SAE, and Observation groups (SBP ≥90 mm Hg)

Variables	Total (n = 6916)	OS (n = 1176)	SAE (n = 1423)	Observation (n = 4317)	p
Age, Median (IQR)	35 (24, 53)	37 (27, 53)	37 (26, 55)	35 (23, 53)	< 0.001
≥55, n (%)	1615 (23.4)	273 (23.2)	358 (25.2)	984 (22.8)	0.187
Sex, n (%)					
Male	4514 (65.3)	793 (67.4)	940 (66.1)	2781 (64.4)	0.123
Female	2402 (34.7)	383 (32.6)	483 (33.9)	1536 (35.6)	
Race, n (%)					
Black	635 (9.2)	95 (8.1)	115 (8.1)	425 (9.8)	0.134
White	5437 (78.6)	945 (80.4)	1142 (80.3)	3350 (77.6)	
Other[a]	734 (10.6)	117 (9.9)	140 (9.8)	477 (11.0)	
Unknow	110 (1.6)	19 (1.6)	26 (1.8)	65 (1.5)	
BMI, n (%)					
<30	4694 (67.9)	838 (71.3)	966 (67.9)	2890 (66.9)	0.028
≥30	1789 (25.9)	260 (22.1)	370 (26.0)	1159 (26.8)	
Unknow	433 (6.3)	78 (6.6)	87 (6.1)	268 (6.2)	
Hospital type, n (%)					
Non-Profit	5993 (86.7)	1039 (88.4)	1216 (85.5)	3738 (86.6)	0.321
Profit	875 (12.7)	132 (11.2)	194 (13.6)	549 (12.7)	
Government	47 (0.7)	5 (0.4)	13 (0.9)	29 (0.7)	
Unknow	1 (0.0)	0 (0.0)	0 (0.0)	1 (0.0)	
Payment, n (%)					
Insurance	3395 (49.1)	550 (46.8)	724 (50.9)	2121 (49.1)	0.241
Medicaid	1348 (19.5)	252 (21.4)	267 (18.8)	829 (19.2)	
Self-Pay	870 (12.6)	169 (14.4)	178 (12.5)	523 (12.1)	
Medicare	742 (10.7)	119 (10.1)	148 (10.4)	475 (11.0)	
Other	471 (6.8)	72 (6.1)	91 (6.4)	308 (7.1)	
Unknow	90 (1.3)	14 (1.2)	15 (1.1)	61 (1.4)	
Bed size, n (%)					
<200	382 (5.5)	60 (5.1)	84 (5.9)	238 (5.5)	0.102
201 to 400	2140 (30.9)	376 (32.0)	423 (29.7)	1341 (31.1)	
401 to 600	1992 (28.8)	356 (30.3)	379 (26.6)	1257 (29.1)	
More than 600	2402 (34.7)	384 (32.7)	537 (37.7)	1481 (34.3)	
Trauma Level, n (%)					
I	3113 (45.0)	557 (47.4)	652 (45.8)	1904 (44.1)	0.002
II	2269 (32.8)	331 (28.1)	456 (32.0)	1482 (34.3)	
III	19 (0.3)	4 (0.3)	2 (0.1)	13 (0.3)	
Unknow	1515 (21.9)	284 (24.1)	313 (22.0)	918 (21.3)	
ED first vital signs					
SBP, Median (IQR)	125 (111, 140)	117 (104,131)	125 (111, 138)	128 (114, 142)	< 0.001
HR, Median (IQR)	90 (77, 103)	93 (80, 111)	88 (76, 102)	89 (77, 102)	< 0.001
≥120, n (%)	600 (8.7)	191 (16.2)	89 (6.3)	320 (7.4)	< 0.001
RR, Median (IQR)	18 (16, 20)	19 (17, 22)	18 (16, 20)	18 (16, 20)	< 0.001
GCS, Median (IQR)	15 (15, 15)	15 (15, 15)	15 (15, 15)	15 (15, 15)	< 0.001

© 2025 Huang W et al. *JAMA Network Open.*

Temperature, n (%)					
<36℃	409 (5.9)	113 (9.6)	81 (5.7)	215 (5.0)	< 0.001
⩾36℃	6068 (87.7)	956 (81.3)	1246 (87.6)	3866 (89.6)	
Unknow	439 (6.3)	107 (9.1)	96 (6.7)	236 (5.5)	
Pulse oximetry, n (%)					
<90%	101 (1.5)	23 (2.0)	11 (0.8)	67 (1.6)	0.013
⩾90%	6756 (97.7)	1140 (96.9)	1394 (98.0)	4222 (97.8)	
Unknow	59 (0.9)	13 (1.1)	18 (1.3)	28 (0.6)	
Respiratory assistance, n (%)					
Yes	110 (1.6)	22 (1.9)	18 (1.3)	70 (1.6)	0.071
No	6696 (96.8)	1142 (97.1)	1389 (97.6)	4165 (96.5)	
Unknow	110 (1.6)	12 (1.0)	16 (1.1)	82 (1.9)	
ISS, Median (IQR)	16 (10, 21)	21 (16, 26)	17 (14, 24)	14 (10, 17)	< 0.001
⩾25, n (%)	1015 (14.7)	480 (40.8)	273 (19.2)	262 (6.1)	< 0.001
Comorbidities, n (%)					
Alcohol Use Disorder	428 (6.2)	96 (8.2)	83 (5.8)	249 (5.8)	0.009
Bleeding Disorder	46 (0.7)	11 (0.9)	13 (0.9)	22 (0.5)	0.122
Chemotherapy for Cancer	23 (0.3)	4 (0.3)	5 (0.4)	14 (0.3)	0.966
Congestive Heart Failure	128 (1.9)	26 (2.2)	18 (1.3)	84 (1.9)	0.154
Smoking	2042 (29.5)	399 (33.9)	427 (30.0)	1216 (28.2)	< 0.001
Chronic Renal Failure	58 (0.8)	5 (0.4)	11 (0.8)	42 (1.0)	0.180
Cerebrovascular Accident	49 (0.7)	8 (0.7)	9 (0.6)	32 (0.7)	0.907
Diabetes Mellitus	500 (7.2)	74 (6.3)	104 (7.3)	322 (7.5)	0.388
Hypertension	1251 (18.1)	208 (17.7)	279 (19.6)	764 (17.7)	0.248
COPD	263 (3.8)	46 (3.9)	56 (3.9)	161 (3.7)	0.919
Steroid Use	33 (0.5)	3 (0.3)	4 (0.3)	26 (0.6)	0.150
Cirrhosis	128 (1.9)	27 (2.3)	30 (2.1)	71 (1.6)	0.245
Dementia	52 (0.8)	5 (0.4)	8 (0.6)	39 (0.9)	0.158
Anticoagulant Therapy	291 (4.2)	59 (5.0)	61 (4.3)	171 (4.0)	0.275
Angina Pectoris	2 (0.0)	1 (0.1)	0 (0.0)	1 (0.0)	0.368
Mental disorder	756 (10.9)	128 (10.9)	165 (11.6)	463 (10.7)	0.659
Myocardial Infarction	19 (0.3)	2 (0.2)	1 (0.1)	16 (0.4)	0.152
Peripheral Arterial Disease	20 (0.3)	2 (0.2)	6 (0.4)	12 (0.3)	0.458
Substance Abuse Disorder	912 (13.2)	210 (17.9)	201 (14.1)	501 (11.6)	< 0.001
Any comorbidities	3830 (55.4)	725 (61.6)	810 (56.9)	2295 (53.2)	< 0.001
Mechanism, n (%)					
MVT	4215 (60.9)	716 (60.9)	895 (62.9)	2604 (60.3)	0.243
Fall	1439 (20.8)	230 (19.6)	282 (19.8)	927 (21.5)	
Other	1262 (18.2)	230 (19.6)	246 (17.3)	786 (18.2)	
Abdominal solid organ AIS, n (%)					
Liver AIS=2	467 (6.8)	94 (8.0)	101 (7.1)	272 (6.3)	0.103
Spleen					
AIS=3	4008 (58.0)	288 (24.5)	533 (37.5)	3187 (73.8)	< 0.001
AIS=4	1893 (27.4)	408 (34.7)	617 (43.4)	868 (20.1)	
AIS=5	1015 (14.7)	480 (40.8)	273 (19.2)	262 (6.1)	
Kidney AIS=2	339 (4.9)	65 (5.5)	60 (4.2)	214 (5.0)	0.294

© 2025 Huang W et al. *JAMA Network Open.*

Pancreas AIS=2	86 (1.2)	42 (3.6)	11 (0.8)	33 (0.8)	< 0.001
AIS of each region, Median (IQR)					
Head	0 (0, 0)	0 (0, 0)	0 (0, 0)	0 (0, 0)	0.180
Face	0 (0, 1)	0 (0, 1)	0 (0, 1)	0 (0, 1)	0.618
Neck	0 (0, 0)	0 (0, 0)	0 (0, 0)	0 (0, 0)	0.289
Chest	0 (0, 2)	0 (0, 2)	0 (0, 2)	0 (0, 2)	0.753
Spine	0 (0, 0)	0 (0, 0)	0 (0, 0)	0 (0, 0)	0.004
Upper extremity	0 (0, 1)	0 (0, 1)	0 (0, 1)	0 (0, 1)	0.001
Lower extremity	0 (0, 1)	0 (0, 1)	0 (0, 1)	0 (0, 1)	< 0.001

BMI, Body Mass Index; SBP, systolic blood pressure; HR, heart rate; RR, respiratory rate; GCS, Glasgow Coma Scale; ISS, injury severity score; AIS, abbreviated injury score; ARDS, acute respiratory distress syndrome; [a] Any races which were not classified as White, Black, or Unknow were classified as other.

© 2025 Huang W et al. *JAMA Network Open.*

eTable 6: outcomes of OS, SAE, and Observation groups (SBP ≥90 mm Hg)

Variables	Total (n = 6916)	OS (n = 1176)	SAE (n = 1423)	Observation (n = 4317)	p
Mortality, n (%)	53 (0.8)	9 (0.8)	12 (0.8)	32 (0.7)	0.929
Failure, n (%)	658 (9.5)	4 (0.3)	89 (6.3)	565 (13.1)	< 0.001
HLOS days, Median (IQR)	5 (4, 7)	6 (5, 9)	5 (4, 7)	5 (3, 7)	< 0.001
ICULOS days, Median (IQR)	3 (2, 4)	3 (2, 5)	3 (2, 4)	3 (2, 4)	< 0.001
Ventilation days, Median (IQR)	2 (2, 4)	2 (2, 3)	2 (2, 3)	2 (2, 4)	0.326
Complications, n (%)					
AKI	33 (0.5)	8 (0.7)	4 (0.3)	21 (0.5)	0.336
ARDS	7 (0.1)	3 (0.3)	3 (0.2)	1 (0.0)	0.014
Cardiac Arrest	26 (0.4)	9 (0.8)	3 (0.2)	14 (0.3)	0.061
Deep SSI	4 (0.1)	2 (0.2)	0 (0.0)	2 (0.0)	0.218
Organ Space SSI	7 (0.1)	6 (0.5)	0 (0.0)	1 (0.0)	< 0.001
Superficial SSI	8 (0.1)	4 (0.3)	1 (0.1)	3 (0.1)	0.058
Severe Sepsis	19 (0.3)	2 (0.2)	3 (0.2)	14 (0.3)	0.727
VTE	54 (0.8)	16 (1.4)	14 (1.0)	24 (0.6)	0.013
DVT	34 (0.5)	10 (0.9)	6 (0.4)	18 (0.4)	0.155
PE	31 (0.4)	9 (0.8)	9 (0.6)	13 (0.3)	0.054
Myocardial Infarction	8 (0.1)	2 (0.2)	2 (0.1)	4 (0.1)	0.664
Stroke	14 (0.2)	2 (0.2)	2 (0.1)	10 (0.2)	0.918
Unplanned Intubation	77 (1.1)	25 (2.1)	15 (1.1)	37 (0.9)	0.001
Unplanned Admission to ICU	167 (2.4)	36 (3.1)	26 (1.8)	105 (2.4)	0.124
Unplanned Visit to OR	100 (1.4)	33 (2.8)	17 (1.2)	50 (1.2)	< 0.001
CAUTI	12 (0.2)	1 (0.1)	2 (0.1)	9 (0.2)	0.843
CLABI	0 (0.0)	0 (0.0)	0 (0.0)	0 (0.0)	1.000
VAP	15 (0.2)	12 (1.0)	1 (0.1)	2 (0.0)	< 0.001
Alcohol Withdrawal Syndrome	41 (0.6)	14 (1.2)	6 (0.4)	21 (0.5)	0.013
Pressure Ulcer	17 (0.2)	8 (0.7)	2 (0.1)	7 (0.2)	0.008
Any Complications	429 (6.2)	126 (10.7)	74 (5.2)	229 (5.3)	< 0.001

HLOS, hospital length of stay; ICULOS, intensive care unit length of stay; AKI, acute kidney injury; ARDS, acute respiratory distress syndrome; SSI, surgical site infection; VTE: venous thromboembolism; DVT, deep vein thrombosis; PE, pulmonary embolism; VAP, ventilator associated pneumonia; CAUTI: catheter associated urinary tract infection; CLABI: central line associated bloodstream infection; OR, operating room.

© 2025 Huang W et al. *JAMA Network Open.*

eTable 7: the baseline of OS, failure of SAE, and failure of Observation groups

Variables	Total (n = 2212)	OS (n = 1499)	SAE (n = 105)	Observation (n = 608)	p
Age, Median (IQR)	41 (28, 57)	39 (28, 56)	44 (30, 62)	45 (30, 59)	0.005
≥55, n (%)	642 (29.0)	403 (26.9)	35 (33.3)	204 (33.6)	0.000
Sex, n (%)					
Male	1431 (64.7)	970 (64.7)	63 (60.0)	398 (65.5)	0.557
Female	781 (35.3)	529 (35.3)	42 (40.0)	210 (34.5)	
Race, n (%)					
Black	191 (8.6)	115 (7.7)	11 (10.5)	65 (10.7)	0.312
White	1787 (80.8)	1218 (81.3)	83 (79.0)	486 (79.9)	
Other[a]	200 (9.0)	141 (9.4)	9 (8.6)	50 (8.2)	
Unknow	34 (1.5)	25 (1.7)	2 (1.9)	7 (1.2)	
BMI, n (%)					
<30	1543 (69.8)	1056 (70.4)	70 (66.7)	417 (68.6)	0.445
≥30	525 (23.7)	344 (22.9)	25 (23.8)	156 (25.7)	
Unknow	144 (6.5)	99 (6.6)	10 (9.5)	35 (5.8)	
Hospital type, n (%)					
Non-Profit	1953 (88.3)	1323 (88.3)	89 (84.8)	541 (89.0)	0.398
Profit	248 (11.2)	169 (11.3)	15 (14.3)	64 (10.5)	
Government	10 (0.5)	7 (0.5)	1 (1.0)	2 (0.3)	
Unknow	1 (0.0)	0 (0.0)	0 (0.0)	1 (0.2)	
Payment, n (%)					
Insurance	982 (44.4)	667 (44.5)	46 (43.8)	269 (44.2)	0.542
Medicaid	477 (21.6)	328 (21.9)	18 (17.1)	131 (21.5)	
Self-Pay	283 (12.8)	203 (13.5)	11 (10.5)	69 (11.3)	
Medicare	307 (13.9)	195 (13.0)	22 (21.0)	90 (14.8)	
Other	133 (6.0)	87 (5.8)	7 (6.7)	39 (6.4)	
Unknow	30 (1.4)	19 (1.3)	1 (1.0)	10 (1.6)	
Bed size, n (%)					
<200	105 (4.7)	73 (4.9)	6 (5.7)	26 (4.3)	0.141
201 to 400	679 (30.7)	476 (31.8)	29 (27.6)	174 (28.6)	
401 to 600	649 (29.3)	453 (30.2)	26 (24.8)	170 (28.0)	
More than 600	779 (35.2)	497 (33.2)	44 (41.9)	238 (39.1)	
Trauma Level, n (%)					
I	1062 (48.0)	711 (47.4)	52 (49.5)	299 (49.2)	0.649
II	640 (28.9)	429 (28.6)	27 (25.7)	184 (30.3)	
III	8 (0.4)	7 (0.5)	0 (0.0)	1 (0.2)	
Unknow	502 (22.7)	352 (23.5)	26 (24.8)	124 (20.4)	
ED first vital signs					
SBP, Median (IQR)	114 (97, 131)	110 (92, 127)	114 (100, 130)	123 (107, 140)	< 0.001
<90, n (%)	382 (17.3)	323 (21.5)	16 (15.2)	43 (7.1)	< 0.001
HR, Median (IQR)	92 (79, 108)	93 (80, 112)	88 (77, 105)	90 (77, 103)	< 0.001
≥120, n (%)	314 (14.2)	248 (16.5)	11 (10.5)	55 (9.0)	< 0.001
RR, Median (IQR)	18 (16, 22)	19 (17, 22)	18 (16, 22)	18 (16, 20)	< 0.001
GCS, Median (IQR)	15 (15, 15)	15 (15, 15)	15 (15, 15)	15 (15, 15)	< 0.001

© 2025 Huang W et al. *JAMA Network Open.*

Temperature, n (%)					< 0.001
<36℃	216 (9.8)	171 (11.4)	8 (7.6)	37 (6.1)	
≥36℃	1789 (80.9)	1170 (78.1)	82 (78.1)	537 (88.3)	
Unknow	207 (9.4)	158 (10.5)	15 (14.3)	34 (5.6)	
Pulse oximetry, n (%)					
<90%	49 (2.2)	42 (2.8)	0 (0.0)	7 (1.2)	0.006
≥90%	2129 (96.2)	1435 (95.7)	100 (95.2)	594 (97.7)	
Unknow	34 (1.5)	22 (1.5)	5 (4.8)	7 (1.2)	
Respiratory assistance, n (%)					
Yes	44 (2.0)	35 (2.3)	1 (1.0)	8 (1.3)	0.462
No	2138 (96.7)	1441 (96.1)	103 (98.1)	594 (97.7)	
Unknow	30 (1.4)	23 (1.5)	1 (1.0)	6 (1.0)	
ISS, Median (IQR)	17 (14, 25)	21 (16, 26)	20 (14, 26)	16 (10, 20)	< 0.001
≥25, n (%)	720 (32.5)	614 (41.0)	38 (36.2)	68 (11.2)	< 0.001
Comorbidities, n (%)					
Alcohol Use Disorder	196 (8.9)	131 (8.7)	8 (7.6)	57 (9.4)	0.808
Bleeding Disorder	29 (1.3)	21 (1.4)	2 (1.9)	6 (1.0)	0.508
Chemotherapy for Cancer	10 (0.5)	6 (0.4)	0 (0.0)	4 (0.7)	0.681
Congestive Heart Failure	58 (2.6)	39 (2.6)	2 (1.9)	17 (2.8)	0.937
Smoking	726 (32.8)	506 (33.8)	31 (29.5)	189 (31.1)	0.379
Chronic Renal Failure	17 (0.8)	8 (0.5)	4 (3.8)	5 (0.8)	0.010
Cerebrovascular Accident	25 (1.1)	16 (1.1)	1 (1.0)	8 (1.3)	0.876
Diabetes Mellitus	170 (7.7)	106 (7.1)	7 (6.7)	57 (9.4)	0.183
Hypertension	457 (20.7)	296 (19.7)	24 (22.9)	137 (22.5)	0.305
COPD	116 (5.2)	70 (4.7)	8 (7.6)	38 (6.2)	0.180
Steroid Use	10 (0.5)	7 (0.5)	0 (0.0)	3 (0.5)	1.000
Cirrhosis	70 (3.2)	49 (3.3)	4 (3.8)	17 (2.8)	0.729
Dementia	16 (0.7)	10 (0.7)	0 (0.0)	6 (1.0)	0.566
Anticoagulant Therapy	129 (5.8)	87 (5.8)	8 (7.6)	34 (5.6)	0.713
Angina Pectoris	1 (0.0)	1 (0.1)	0 (0.0)	0 (0.0)	1.000
Mental disorder	268 (12.1)	171 (11.4)	17 (16.2)	80 (13.2)	0.227
Myocardial Infarction	8 (0.4)	5 (0.3)	0 (0.0)	3 (0.5)	0.789
Peripheral Arterial Disease	11 (0.5)	10 (0.7)	0 (0.0)	1 (0.2)	0.351
Substance Abuse Disorder	392 (17.7)	268 (17.9)	17 (16.2)	107 (17.6)	0.905
Any comorbidities	1401 (63.3)	950 (63.4)	65 (61.9)	386 (63.5)	0.951
Mechanism, n (%)					
MVT	1226 (55.4)	859 (57.3)	50 (47.6)	317 (52.1)	0.029
Fall	555 (25.1)	354 (23.6)	37 (35.2)	164 (27.0)	
Other	431 (19.5)	286 (19.1)	18 (17.1)	127 (20.9)	
Abdominal solid organ AIS, n (%)					
Liver AIS=2	163 (7.4)	123 (8.2)	7 (6.7)	33 (5.4)	0.083
Spleen					
AIS=3	738 (33.4)	372 (24.8)	31 (29.5)	335 (55.1)	< 0.001
AIS=4	754 (34.1)	513 (34.2)	36 (34.3)	205 (33.7)	
AIS=5	720 (32.5)	614 (41.0)	38 (36.2)	68 (11.2)	
Kidney AIS=2	90 (4.1)	72 (4.8)	3 (2.9)	15 (2.5)	0.039

© 2025 Huang W et al. *JAMA Network Open.*

Pancreas AIS=2	56 (2.5)	49 (3.3)	1 (1.0)	6 (1.0)	0.004
AIS of each region, Median (IQR)					
Head	0 (0, 0)	0 (0, 0)	0 (0, 0)	0 (0, 0)	0.372
Face	0 (0, 0)	0 (0, 1)	0 (0, 0)	0 (0, 0)	0.321
Neck	0 (0, 0)	0 (0, 0)	0 (0, 0)	0 (0, 0)	0.504
Chest	0 (0, 1)	0 (0, 2)	0 (0, 1)	0 (0, 1)	0.327
Spine	0 (0, 0)	0 (0, 0)	0 (0, 0)	0 (0, 0)	0.012
Upper extremity	0 (0, 1)	0 (0, 1)	0 (0, 0)	0 (0, 1)	0.591
Lower extremity	0 (0, 1)	0 (0, 1)	0 (0, 1)	0 (0, 1)	0.213

BMI, Body Mass Index; SBP, systolic blood pressure; HR, heart rate; RR, respiratory rate; GCS, Glasgow Coma Scale; ISS, injury severity score; AIS, abbreviated injury score; ARDS, acute respiratory distress syndrome; [a] Any races which were not classified as White, Black, or Unknow were classified as other.

© 2025 Huang W et al. *JAMA Network Open.*

eTable 8: outcomes of OS, failure of SAE, and failure of Observation groups

Variables	Total (n = 2212)	OS (n = 1499)	SAE (n = 105)	Observation (n = 608)	p
Mortality, n (%)	44 (2.0)	29 (1.9)	6 (5.7)	9 (1.5)	0.037
HLOS days, Median (IQR)	35 (1.6)	20 (1.3)	4 (3.8)	11 (1.8)	0.100
ICULOS days, Median (IQR)	7 (5, 10)	6 (5, 9)	10 (7, 13)	7 (5, 10)	< 0.001
Ventilation days, Median (IQR)	3 (2, 5)	3 (2, 5)	4 (3, 6)	3 (2, 5)	< 0.001
Complications, n (%)	2 (2, 3)	2 (2, 3)	2.5 (2, 4)	2 (2, 4)	0.042
AKI					
ARDS	38 (1.7)	24 (1.6)	5 (4.8)	9 (1.5)	0.075
Cardiac Arrest	8 (0.4)	6 (0.4)	2 (1.9)	0 (0.0)	0.030
Deep SSI	27 (1.2)	19 (1.3)	2 (1.9)	6 (1.0)	0.577
Organ Space SSI	4 (0.2)	4 (0.3)	0 (0.0)	0 (0.0)	0.643
Superficial SSI	8 (0.4)	7 (0.5)	0 (0.0)	1 (0.2)	0.627
Severe Sepsis	4 (0.2)	4 (0.3)	0 (0.0)	0 (0.0)	0.661
VTE	20 (0.9)	10 (0.7)	3 (2.9)	7 (1.2)	0.055
DVT	24 (1.1)	13 (0.9)	3 (2.9)	8 (1.3)	0.098
PE	16 (0.7)	10 (0.7)	1 (1.0)	5 (0.8)	0.663
Myocardial Infarction	5 (0.2)	4 (0.3)	1 (1.0)	0 (0.0)	0.143
Stroke	5 (0.2)	2 (0.1)	0 (0.0)	3 (0.5)	0.340
Unplanned Intubation	52 (2.4)	32 (2.1)	4 (3.8)	16 (2.6)	0.392
Unplanned Admission to ICU	117 (5.3)	47 (3.1)	12 (11.4)	58 (9.5)	< 0.001
Unplanned Visit to OR	111 (5.0)	48 (3.2)	21 (20.0)	42 (6.9)	< 0.001
CAUTI	4 (0.2)	3 (0.2)	0 (0.0)	1 (0.2)	1.000
CLABI	0 (0.0)	0 (0.0)	0 (0.0)	0 (0.0)	1.000
VAP	16 (0.7)	14 (0.9)	1 (1.0)	1 (0.2)	0.118
Alcohol Withdrawal Syndrome	25 (1.1)	19 (1.3)	1 (1.0)	5 (0.8)	0.749
Pressure Ulcer	15 (0.7)	12 (0.8)	0 (0.0)	3 (0.5)	0.776
Any Complications	324 (14.6)	175 (11.7)	39 (37.1)	110 (18.1)	< 0.001

HLOS, hospital length of stay; ICULOS, intensive care unit length of stay; AKI, acute kidney injury; ARDS, acute respiratory distress syndrome; SSI, surgical site infection; VTE: venous thromboembolism; DVT, deep vein thrombosis; PE, pulmonary embolism; VAP, ventilator associated pneumonia; CAUTI: catheter associated urinary tract infection; CLABI: central line associated bloodstream infection; OR, operating room.

© 2025 Huang W et al. *JAMA Network Open.*
