# Supplementary material for: Outcomes of Isolated Severe Blunt Splenic Injury
Source: JAMA Netw Open. 2025 Sep 23;8(9):e2533266. doi: 10.1001/jamanetworkopen.2025.33266 (PMC12457976; doi:10.1001/jamanetworkopen.2025.33266)
Supplement: Supplement 2. — Data Sharing Statement [file jamanetwopen-e2533266-s002.pdf]

Data Sharing Statement

Huang. Outcomes of Isolated Severe Blunt Splenic Injury. *JAMA Netw Open*. Published September 23, 2025. doi:10.1001/jamanetworkopen.2025.33266

Data
Data available: No
